# Drivers to adopting B-flow ultrasonography:contextualizing the integrated technology acceptance model

**DOI:** 10.1186/s12880-019-0356-y

**Published:** 2019-07-12

**Authors:** Gulsah Hancerliogullari Koksalmis

**Affiliations:** 1 Department of Industrial Engineering, Faculty of Management, Istanbul Technical University, Istanbul, Turkey; 2 School of Management, Centre of Operational Research, Management Sciences & Information Systems, University of Southampton, Southampton, UK

**Keywords:** Ovarian torsion, B-flow ultrasonography (B-flow USG), Technology acceptance model, Knowledge, Social influence

## Abstract

**Background:**

Ovarian torsion is an unprecedented gynecological crisis that regularly influences ladies of regenerative age. Its signs and indications are like those of other abdominal conditions, which make its differential determination testing. B-flow ultrasonography (B-flow USG), which is utilized for the differential determination of ovarian torsion, is the highest quality level non-intrusive indicative instrument in the early period of an ovarian torsion. The aim of this paper is to investigate and incorporate variety of factors affecting physicians’ actual use of B-flow USG.

**Methods:**

Drawing from technology acceptance model (TAM), five variables – actual use, behavioral intention to use, attitude toward use, perceived ease of use and perceived usefulness – are integrated with social influence and knowledge to propose a theoretical model. The data was collected from the medical doctors including radiologists, urologists, gynecologists, pediatric surgeons between June and October 2018. The sample size is *N* = 512 hence, structural equation modeling (SEM) methodology has been implemented to study the relationship between explanatory factors and actual use of B-flow USG. SmartPLS 3.2.7 software was used for the data analysis and testing of the validity of the eight hypotheses.

**Results:**

The results indicate that actual use of B-flow USG is positively affected by knowledge, social influence, perceived ease of use, perceived usefulness, attitude toward use and behavioral intention to use.

**Conclusions:**

It is discovered that perceived usefulness mediates the relationship between perceived ease of use and attitude toward use, and attitude toward use mediates the relationship between perceived usefulness and behavioral intention to use B-flow USG. The implications of the outcomes are discussed, and suggestions for future research are made.

## Background

Ovarian torsion is a surprising gynecological crisis that comprises around 2.7% of the gynecologic protestations in regenerative age ladies [1–3]. The most well-known reasons for ovarian torsion, which happens because of the turn of the pedicle of the ovarian tissue, are irritation and injury [3]. Ovarian torsion regularly gives nonspecific manifestations, including abdominal torment, spasms, testiness, loss of craving and hot flashes [2, 4]. In view of these nonspecific manifestations, the differential finding of the infection from other abdominal conditions is testing. Past investigations have announced that exploratory laparotomy, which is regularly performed dependent on preservationist demonstrative techniques, has a 56% false conclusion rate [4]. Numerous investigations have detailed that an exact conclusion must be made by visual finding amid an obtrusive laparoscopy or laparotomy [2]. With the ongoing advances in the innovation, the utilization of non-intrusive analytic apparatuses is obligatory. What’s more, it is vital to assess the pedicle blood flow and blood divider structure for the conclusion of the infection with the end goal to counteract superfluous medical procedure. While color Doppler USG has been generally utilized for the differential finding of ovarian torsion throughout the previous 2 years, the as of late presented B-flow ultrasonography imaging framework seems to conquer the challenges in the assessment of blood flow and divider thickness. B-flow is another strategy that expands the goals, outline rate and dynamic scope of B-mode to at the same time picture blood flow and tissue [5–7].

Despite the increasing usage of B-flow USG, research on actual use of B-flow USG is scarce. The aim of this empirical study is to fill this gap by exploring the acceptance of B-flow USG, and various factors that affect physicians’ preference of B-flow USG use. None of the empirical research takes into account both drivers and barriers to predict user intention to adopt B-flow USG. So as to have a robust theoretical basis for investigating the acceptance of B-flow USG, this study draws on technology acceptance model which has been utilized in numerous researches to anticipate and find out user perception of system use.

The contributions of this study are fourfold. First, this study identifies which determinants are more effective in acceptance of B-flow USG use. No previous research demonstrates factors affecting the adoption of B-flow USG. Moreover, there has been no study where the constructs used in this model are modeled together to determine the determinants of actual use of B-flow USG. Second, there are few researches studied the determinants of actual use of medical systems; however, to the best of knowledge, the current research has the largest sample size with 528 participants and more than 97% of the responses were used in the analysis. Third, this study assesses if the incorporation of technology acceptance model supplies a robust theoretical basis for studying the acceptance of B-flow USG. This paper discovered two mediation effects: perceived usefulness mediates the relationship between perceived ease of use and attitude toward use, besides attitude toward use mediates the relationship between perceived usefulness and behavioral intention to use B-flow USG. Fourth, the results of this study prove that behavioral intention to use is significantly and directly associated to actual use of B-flow USG. Knowledge, social influence, perceived ease of use, perceived usefulness and attitude toward use also influence actual use of B-flow USG significantly and indirectly through behavioral intention to use. The results of the present study are of high relevance to stakeholders including B-flow USG users, hospital managements, manufacturers and suppliers. The target audience also involves business analysts and researchers who seek to comprehend the factors driving physicians to use B-flow USG and, hence, to make future forecasts of the imaging technology and its distribution.

The organization of the rest of the paper is as follows: In the second section theoretical background and hypotheses are provided. The research methodology is explained in the third section. Next, the results of the analysis are provided. The paper concludes with discussion of the findings, directions for future research and theoretical and practical implications.

### Theoretical background and hypotheses development

**Fig. 1:**
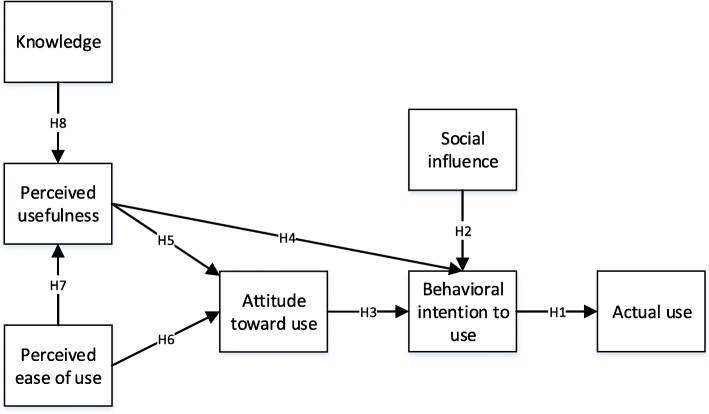
Proposed research model

The theoretical basis of this research consists of Technology Acceptance Model. So as to investigate the acceptance of a novel technology, B-flow USG, the integration of this model with knowledge and social influence is selected as a theoretical framework. The proposed research framework is shown in Fig. 1. There are 7 constructs, specifically, actual use, attitude toward use, behavioral intention to use, knowledge, perceived ease of use, perceived usefulness, social influence with 32 items. The hypotheses are developed based on the literature.

#### Technology acceptance model

The technology acceptance model is currently a common predictive tool for testing the users’ acceptance of new technologies in various industries including healthcare, education, e-commerce, etc. [8]. TAM, which describes users’ behavior towards information technology adoption, is a model emerging from theory of reasoned action (TRA) [9]. TAM consists of the following factors, i.e., constructs, which can be utilized to solve any technology acceptance problems: actual system use, behavioral intention to use, attitude towards use, perceived usefulness, perceived ease of use and external variables. According to TAM, which is an information systems theory, perceived usefulness (PU) and perceived ease of use (PEU) are the primary antecedents of intention to use information technology. Moreover, it was indicated that perceived ease of use influences perceived usefulness and attitude towards use; perceived usefulness affects both attitude towards use and behavioral intention to use; attitude towards use influence behavioral intention to use which determines actual system use.

### Actual use

Actual use is a measure of the frequency and intensity of the use of information technology in an individual’s work. The level of use of the new technology shows the use of the system and the acceptance of the technology. To get more benefits from the B-flow USG, surely the system usage needs to increase. So, this helps the physicians to achieve their goals for execution of B-flow USG [10].

### Behavioral intention to use

Behavioral intention means “the degree to which a person has formulated conscious plans to perform or not perform some specified future behavior” [11]. It is defined as “a behavioral tendency of people to keep using a certain technology” [8]. It is a determinant of the possibility that an individual will be engaged in a certain behavior [12]. The more a person is willing to use a system, the higher is his or her likelihood of using it [13]. In the current study, since the usage of B-flow USG is done of one’s own fee will, finding out the determinants of behavioral intention to use B-flow USG is important for the actual use of it. Earlier work have described the impact of behavioral intention on actual use. As a result, it was hypothesized as follows:

- *H1. Behavioral intention has a positive relationship with actual use of* B-flow USG*.*

### Social influence

Social influence refers to “a person’s perception that most people who are import to him think he should or should not perform the behavior in question” [14]. Social influence, which affects the behavioral intention directly is denoted as subjective norm in TRA and Extended TAM, and it is represented as social factors in Model of PC Utilization. Social influence is crucial merely in the initial periods of personal experimentation with the technology. It is indicated that social influence affects behavioral intention positively [11, 15]. Users will intend to use B-flow USG if their families, friends and peers use it or recommend using it. As a result, it was hypothesized as follows:

- *H2. Social influence has a positive relationship with behavioral intention to use* B-flow USG*.*

### Attitude toward use

Attitude refers to “individual’s positive or negative feelings about performing the target behavior” [14, 16]. In other words, it is the person’s behavior for a service or a product. Based on the TRA, behavioral intention to use is affected by the attitude toward behavior and subjective norms. In accordance with the TAM, perceived usefulness and attitude toward use determine the behavioral intention to use. Several researches have shown the impact of attitude toward use on behavioral intention to use, which is significant [15, 17–20]. As a result, it was hypothesized as follows:

- *H3. Attitude toward use has a positive relationship with behavioral intention to use* B-flow USG*.*

### Perceived usefulness

Perceived usefulness is “the degree to which an individual believes that using a particular system would enhance his or her job performance” [8]. Put differently, it means that B-flow USG usage will improve the productivity of the user and ultimately result in being more efficient and accurate [21]. In the original technology acceptance model, perceived usefulness is a key factor affecting behavioral intention to use [16]. Several researches in various areas verifies the effect of perceived usefulness on the behavioral intention to use and attitude toward use [18, 22–24]. Thus, in addition to various application areas, the effect of perceived usefulness on behavioral intention to use B-flow USG may be critical. As a result, it was hypothesized as follows:

- *H4. Perceived usefulness has a positive relationship with behavioral intention to use* B-flow USG*.*

In the original TAM, not only perceived usefulness but also perceived ease of use are affecting attitude toward use. Moreover, perceived usefulness and attitude toward use affect behavioral intention to use [8]. A user who thinks that B-flow USG usage improves his or her performance has an optimistic attitude toward use B-flow USG. Several research have demonstrated the positive and significant relationship between perceived usefulness and attitude toward use [8, 18, 25, 26]. Hence, it was hypothesized as follows:

- *H5. Perceived usefulness has a positive relationship with attitude toward use* B-flow USG*.*

### Perceived ease of use

Perceived ease of use is “the degree to which a person believes that using a particular system would be free of effort” [8]. Alias, it is a sign of the intellectual strength necessary for learning and using an information system [27]. Both perceived usefulness and perceived ease of use are affecting attitude toward use in the original TAM. A user who thinks that using the B-flow USG would be easy has an optimistic attitude toward use B-flow USG. Therefore, it was hypothesized as follows:

- *H6. Perceived ease of use has a positive relationship with attitude toward use* B-flow USG*.*

If all other factors are held constant, the easier the system, the higher the user’s job performance is, the more useful it can be [28]. Users may not feel confident with a new system, since they do not have the necessary skills and comfort. Nevertheless, after gaining a certain amount of knowledge and being more familiar with it, their perception of its ease of use may change [29]. If using B-flow USG is easy, people may not need to waste time to find out how to use them. Research in various fields has confirmed the significant effect of perceived ease of use on perceived usefulness [11, 18, 19, 25, 30]. Thus, it was hypothesized as follows:

- *H7. Perceived ease of use has a positive relationship with perceived usefulness of* B-flow USG*.*

### Knowledge

Understanding of a technology is considered to be a learned behavior according to the Theory of Reasoned Action [14]. Therefore, it ought to track the growth of knowledge via experimentation. Although several TAM studies evaluate user knowledge directly, there are many research have utilized experimentation to represent knowledge [31]. the following research mainly studies practical knowledge, through evaluating users’ estimations of their individual comparative expertise with technology [32]. Earlier research has verified that the knowledge has a positive effect on perceived usefulness [33]. Therefore, it was hypothesized as follows:

- *H8. Knowledge has a positive relationship with perceived usefulness of* B-flow USG*.*

## Methods

### Survey design

This study depends on information collected from B-flow USG users. A survey methodology was conducted to collect the data and to validate the conceptual model proposed in this research. The sample was drawn from the medical doctors including radiologists, urologists, gynecologists, pediatric surgeons.

The survey consisted of three parts. The first part included the cover letter and informed consent statement. The second part consisted of the questions related to demographics, such as age, gender, B-flow USG experience, etc.. The third part consisted of the questions related to the TAM and additional items assessing knowledge and social influence. The survey did not contain any personal information that could potentially specify the identity of a particular respondent.

### Data collection

For the aim of the research, the data was collected through online surveys. The objective of the study and background on the research were provided to the participants, and they are encouraged to participate. So as to evaluate the acceptance of B-flow USG, a survey of all users was preferred.

A total of 528 surveys were collected from participants, and 512 of them were used in the analysis, which is adequate since, as a rule of thumb, the sample size required for factor analysis should be at least four or five times of the number of items [34]. Overall, 67.5% of the respondents were male, and the average age of the participants was 36.5. Of the respondents, 59% of them is working at university hospitals and average B-flow USG experience is 6.2 years. The summary of demographic information of the participants is provided in Table 1.

**Table 1:** Demographic information

*Age (years)*		
Max: 60	Min: 26	Avg: 36.5
*Gender (%)*		
Female: 32.5	Male: 67.5	
*Workplace (%)*		
Private Hospital: 4.2	University Hospital: 59.5	Training and Research Hospital: 22.2
Public Hospital: 14.1		
*Work experience as an MD (years)*		
Max: 35	Min: 2	Avg: 19.3
*B-flow ultrasonography experience (%)*		
Max: 10	Min: 2	Avg: 6.2

### Measurement development

The five-point Likert scale was utilized to measure the variables in this paper. There are 7 constructs with 32 items. The three items for the actual use (e.g., AU01 AU02, AU03) were taken from [35]. The construct attitude toward use was measured by four items (e.g., ATT01, ATT02, ATT03, ATT04) from [36]. The three items to measure the construct behavioral intention to use (e.g., BIU01, BIU02, BIU03) were taken from [36]. The construct knowledge was measured by three items (e.g., KNOW01, KNOW02, KNOW03) from [33]. The six items for the construct perceived ease of use (e.g., PEU01, PEU2, …, PEU06) were taken from [8, 16, 36]. The seven items to measure the construct perceived usefulness (e.g., PU01, PU02, …, PU07) were taken from [8, 16, 36]. The six items for the construct social influence (e.g., SI01, SI02, …, SI06) were taken from [36, 37]. The details of the constructs and items are provided in Table 2.

**Table 2:** Constructs, codes, references and items

Construct	Item Code	Reference	Items
Actual use	AU1	[35]	“I use the B-flow USG very intensively (many hours per day, at work)”
	AU2		“I use the B-flow USG very frequently (many times per day, at work)”
	AU3		“Overall, I use the B-flow USG a lot”
Attitude toward use	ATT01	[36]	“Using the B-flow USG is a good idea.”
	ATT02		“The B-flow USG makes work more interesting.”
	ATT03		“Working with the B-flow USG is fun.”
	ATT04		“I like working with the B-flow USG.”
Behavioral intention to use	BIU01	[36]	“I intend to use the B-flow USG in the next <n > months.”
	BIU02		“I predict I would use the B-flow USG in the next <n > months.”
	BIU03		“I plan to use the B-flow USG in the next <n > months.”
Knowledge	KNOW01	[33]	“I have knowledge about the B-flow USG.”
	KNOW02		“I have knowledge about the benefits of the B-flow USG.”
	KNOW03		“I have knowledge about the B-flow USG know-how.”
Perceived ease of use	PEU01	[8, 16, 36]	“Learning to operate the B-flow USG would be easy for me.”
	PEU02		“I would find it easy to get the B-flow USG to do what I want it to do.”
	PEU03		“My interaction with the B-flow USG would be clear and understandable.”
	PEU04		“I would find the B-flow USG to be flexible to interact with.”
	PEU05		“It would be easy for me to become skillful at using the B-flow USG.”
	PEU06		“I would find the B-flow USG easy to use.”
Perceived usefulness	PU01	[8, 16, 36]	“Using the B-flow USG would improve my productivity.”
	PU02		“Using the B-flow USG would increase my efficiency in transaction.”
	PU03		“Using the B-flow USG would make my transaction easier.”
	PU04		“Using the B-flow USG would make my transaction quicker.”
	PU05		“I find the B-flow USG to be cost saving.”
	PU06		“I think the B-flow USG is valuable to me.”
	PU07		“Overall, I find the B-flow USG to be useful.”
Social influence	SI01	[36, 37]	“People who influence my behavior think that I should use the B-flow USG.”
	SI02		“People who are important to me think that I should use the B-flow USG.”
	SI03		“I use the B-flow USG because of the proportion of coworkers who use the B-flow USG.”
	SI04		“People in my organization who use the B-flow USG have more prestige than those who do not.”
	SI05		“People in my organization who use the B-flow USG have a high profile.”
	SI06		“Having the B-flow USG is a status symbol in my organization.”

### Data analysis

In this study, a multivariate analysis method, specifically, the partial least squares structural equation modeling (PLS-SEM), is used to test and validate the proposed model. This modeling approach enables researchers to explicate relatively new circumstance from measures and models even in the lack of theoretical background. The PLS-SEM, which was used in similar research in the literature, provides accurate estimations even the data is not normally distributed [38]. The SmartPLS 3.2.7 software was used to perform the PLS-SEM technique and the do the analyses.

## Results

### Measurement model

In order to test the measurement model’s psychometric features, confirmatory factor analysis (CFA) was performed. The construct reliability and validity of the constructs were evaluated reviewing the content validity, convergent validity and discriminant validity of the constructs. The content validity was evaluated through corresponding literature and pilot testing the scale [39]. The convergent validity was assessed by item reliability of each measure, Cronbach’s alpha, composite reliability (CR) of each construct and the average variance extracted (AVE) [39–41].

The item reliability of a measure is evaluated by its factor loadings which represent the correlation between each item and the respective construct. The recommended values for factor loadings is at least 0.6 [34, 41]. As provided in Table 3, the factor loadings of all the items are above 0.7 which proves convergent validity at the item level; therefore, none of the items had to be removed from the proposed research model. Moreover, the results show that all of the items have significant t-statistics (*p* < 0.05).

**Table 3:** Confirmatory factor analysis

Construct	Item Code	Mean	Standard Deviation	Factor Loadings	T Statistics
Attitude toward use	ATT01	0.922	0.005	0.922	185.473
	ATT02	0.814	0.014	0.813	59.105
	ATT03	0.774	0.017	0.773	46.311
	ATT04	0.801	0.015	0.802	54.936
Actual use	AU01	0.927	0.004	0.926	212.943
	AU02	0.834	0.013	0.833	63.876
	AU03	0.845	0.013	0.846	65.702
Behavioral intention to use	BIU01	0.933	0.004	0.933	233.299
	BIU02	0.836	0.011	0.837	73.593
	BIU03	0.856	0.011	0.857	80.727
Knowledge	KNOW01	0.925	0.004	0.924	220.230
	KNOW02	0.825	0.014	0.825	56.960
	KNOW03	0.830	0.015	0.830	53.583
Perceived ease of use	PEU01	0.924	0.004	0.924	221.790
	PEU02	0.784	0.017	0.784	46.833
	PEU03	0.783	0.016	0.783	48.758
	PEU04	0.767	0.018	0.767	42.854
	PEU05	0.758	0.018	0.758	42.149
	PEU06	0.770	0.017	0.770	44.367
Perceived usefulness	PU01	0.927	0.004	0.928	236.571
	PU02	0.749	0.017	0.749	43.990
	PU03	0.773	0.016	0.772	48.889
	PU04	0.742	0.017	0.743	43.684
	PU05	0.770	0.017	0.771	45.973
	PU06	0.751	0.017	0.749	43.880
	PU07	0.761	0.016	0.762	46.390
Social influence	SI01	0.927	0.004	0.927	222.203
	SI02	0.744	0.017	0.746	42.618
	SI03	0.782	0.016	0.781	50.388
	SI04	0.730	0.019	0.731	39.198
	SI05	0.765	0.017	0.766	44.741
	SI06	0.777	0.016	0.777	49.935

At the construct level, the reliability was evaluated by measuring the Cronbach’s alpha and composite reliability values (Table 4). The Cronbach’s alpha quantifies the level of internal consistency of constructs, and the higher is the better. It was suggested that Cronbach’s alpha values above 0.7 should be considered good [41]. In this study, the Cronbach’s alpha values of each construct exceed the threshold, which implies that each of the construct has a good internal consistency. The composite reliability denotes “the shared variance among a set of observed variables measuring an underlying construct” [41]; in other words, how well the items measure a construct. The CR values for the corresponding measures varied between 0.895 and 0.917, exceeding the recommended value of 0.7 [41, 42].

**Table 4:** Construct reliability and validity for the measurement model

Constrtuct	Cronbach’s Alpha	Composite Reliability (CR)	Average Variance Extracted (AVE)
ATT	0.848	0.898	0.688
AU	0.839	0.903	0.756
BIU	0.849	0.909	0.769
KNOW	0.826	0.895	0.741
PEU	0.886	0.914	0.640
PU	0.894	0.917	0.615
SI	0.878	0.909	0.625

Average variance extracted is the last measure of convergent validity; it indicates “the variance captured by the construct in relation to the amount of variance attributable to measurement error”, and the recommended value for AVE is greater than 0.5 [41, 43]. The results indicate that the AVE values of all constructs are greater than the recommended value 0.5 (Table 4). The statistical results imply good convergent validity of the measurement model.

Next, the discriminant validity was assessed which measures the extent to which constructs differ. Discriminant validity at the item level exists “when an item correlates more highly with items in the construct it purports to measure than items belonging to other constructs” [44, 45], which is also valid for this study. Discriminant validity of the measurement constructs are evaluated through “comparing the square root of the AVE for a given construct with the correlations between that construct and all other constructs” [46]. The correlation matrix for the constructs are provided in Table 5. Discriminant validity at the construct level appears satisfactory since the diagonal elements, square roots of the AVEs, are larger than the off-diagonal elements in their corresponding rows and columns.

**Table 5:** Discriminant validity for the measurement model

	ATT	AU	BIU	KNOW	PEU	PU	SI
ATT	0.830						
AU	0.513	0.869					
BIU	0.675	0.673	0.877				
KNOW	0.439	0.221	0.279	0.861			
PEU	0.348	0.187	0.217	0.458	0.800		
PU	0.602	0.327	0.432	0.649	0.593	0.784	
SI	0.492	0.458	0.670	0.230	0.123	0.302	0.791

### Structural model and hypotheses testing

Structural equation modeling is performed to check the fit between the proposed research model and the data. The reason for applying this methodology is to observe several relationships simultaneously, particularly where there are direct and indirect effects among the constructs within the model [34].

PLS-SEM through SmartPLS 3.2.7 was used in order to test the hypotheses. Non-parametric assessment criteria based on bootstrapping is performed with 5000 iterations of resampling [47, 48]. Table 6 summarizes the standardized parameters (β coefficients) and the corresponding t-values to measure each coefficient’s significance [38].

**Table 6:** Path coefficients

	Standardized Path Coefficient (β Coefficient)	Standard Deviation	T Statistics	*P* Values
ATT - > BIU	0.435^*^	0.036	11.935	0.000
BIU - > AU	0.673^*^	0.020	34.386	0.000
KNOW - > PU	0.478^*^	0.028	17.316	0.000
PEU - > ATT	0.015	0.047	0.312	0.755
PEU - > PU	0.374^*^	0.029	12.805	0.000
PU - > ATT	0.611^*^	0.036	17.147	0.000
PU - > BIU	0.036	0.037	0.980	0.328
SI - > BIU	0.445^*^	0.030	14.925	0.000

^*^p < 0.05

Behavioral intention to use significantly affects actual use of B-flow USG (β = 0.673, *p* < 0.05; H1 supported). Social influence and attitude toward use significantly affect behavioral intention to use B-flow USG (β = 0.445, *p* < 0.05; β = 0.435, *p* < 0.05; H2 and H3 supported); however, the effect of perceived usefulness on behavioral intention to use B-flow USG is found to be insignificant (β = 0.036, *p* > 0.05; H4 not supported). Perceived usefulness significantly influences attitude toward use B-flow USG (β = 0.611 *p* < 0.05; H5 supported); on the other hand, and perceived ease of use affects attitude toward use B-flow USG, but not significantly (β = 0.015, *p* > 0.05; H6 not supported). The effects of and perceived ease of use and knowledge on perceived usefulness are significant (β = 0.374, *p* < 0.05; β = 0.478, *p* < 0.05; H7 and H8 supported). All the hypotheses which are supported or not are summarized in Table 7.

**Table 7:** Summary of hypotheses

Hypothesis	Path	Hypothesis	Supported
H1	BIU - > AU	“Behavioral intention to use has a positive effect on actual use of B-flow USG.”	Yes
H2	SI - > BIU	“Social influence has a positive effect on behavioral intention to use B-flow USG.”	Yes
H3	ATT - > BIU	“Attitude toward use has a positive effect on behavioral intention to use B-flow USG.”	Yes
H4	PU - > BIU	“Perceived usefulness has a positive effect on behavioral intention to use B-flow USG.”	No
H5	PU - > ATT	“Perceived usefulness has a positive effect on attitude toward use B-flow USG.”	Yes
H6	PEU- > ATT	“Perceived ease of use has a positive effect on attitude toward use B-flow USG.”	No
H7	PEU - > PU	“Perceived ease of use has a positive effect on perceived usefulness of B-flow USG.”	Yes
H8	KNOW- > PU	“Knowledge has a positive effect on perceived usefulness of B-flow USG.”	No

The R-square (R^2^) value specifies the percentage of total variance of the dependent variable (i.e., actual use) explained by the independent variables. Among the endogenous constructs, behavioral intention to use has the highest amount of variance accounted by its exogenous construct, 61% (R^2^ = 0.607). The results further show that proposed model explains the 36% (R^2^ = 0.363) of total variance of attitude toward use B-flow USG, 45% (R^2^ = 0.453) of total variance of actual use, 53% (R^2^ = 0.532) of total variance of perceived usefulness, as provided in Table 8.

**Table 8:** R-Square values

	R-Square	R-Square (Adjusted)
ATT	0.363	0.360
AU	0.453	0.452
BIU	0.607	0.605
PU	0.532	0.530

**Fig. 2:**
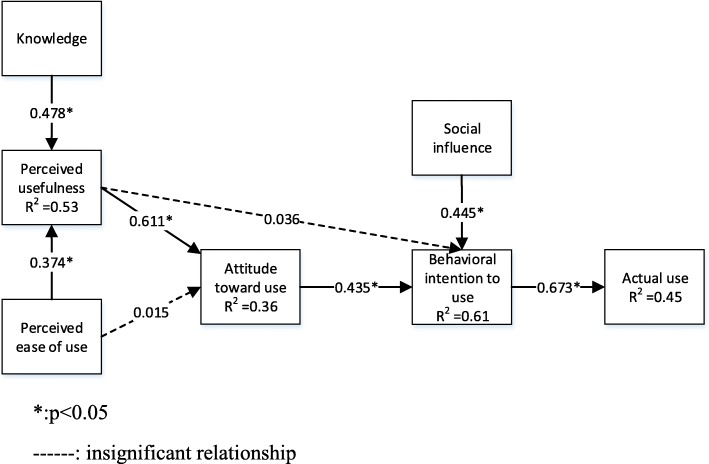
Path coefficients of the research model

Figure 2 illustrates the β coefficients and the R^2^ values of the dependent variables. The analysis implies that behavioral intention to use is a significant determinant of actual use of B-flow USG. It is shown that behavioral intention to use is explained by social influence and attitude toward use, which is consistent with the earlier literature; nevertheless, the effect of perceived usefulness on behavioral intention to use B-flow USG is found to be insignificant. Perceived usefulness significantly influences attitude toward use B-flow USG; on the other hand, in contrast to literature, perceived ease of use affects attitude toward use insignificantly. Finally, perceived ease of use and knowledge are found to be significant determinants of perceived usefulness.

### Direct and indirect effects

Direct, indirect and total effects of each construct on the behavioral intention to use B-flow USG are provided in Table 9. Behavioral intention to use has the highest direct and total effect on actual use of B-flow USG. Attitude toward use, knowledge, perceived ease of use, perceived usefulness and social influence have significant indirect effects on actual use of B-flow USG.

**Table 9:** Direct, indirect and total effects on actual use of B-flow USG

Dependent Variable	Independent Variables	Direct Effects	Indirect Effects	Total Effects	*P* Values
Actual use of B-flow USG	ATT	–	0.293^*^	0.293^*^	0.000
	BIU	0.673^*^	–	0.673^*^	0.000
	KNOW	–	0.097^*^	0.097^*^	0.000
	PEU	–	0.072^*^	0.072^*^	0.000
	PU	–	0.203^*^	0.203^*^	0.000
	SI	–	0.300^*^	0.300^*^	0.000

*p < 0.05

**Fig. 3:**
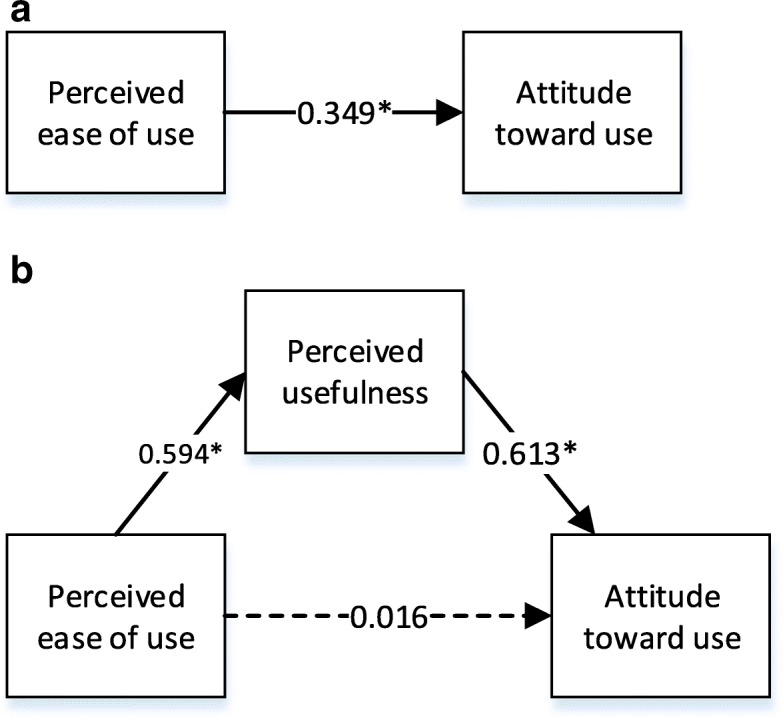
**a** Standardized parameters of direct effects. **b** Standardized parameters of indirect effects (mediation effect of perceived usefulness)

**Fig. 4:**
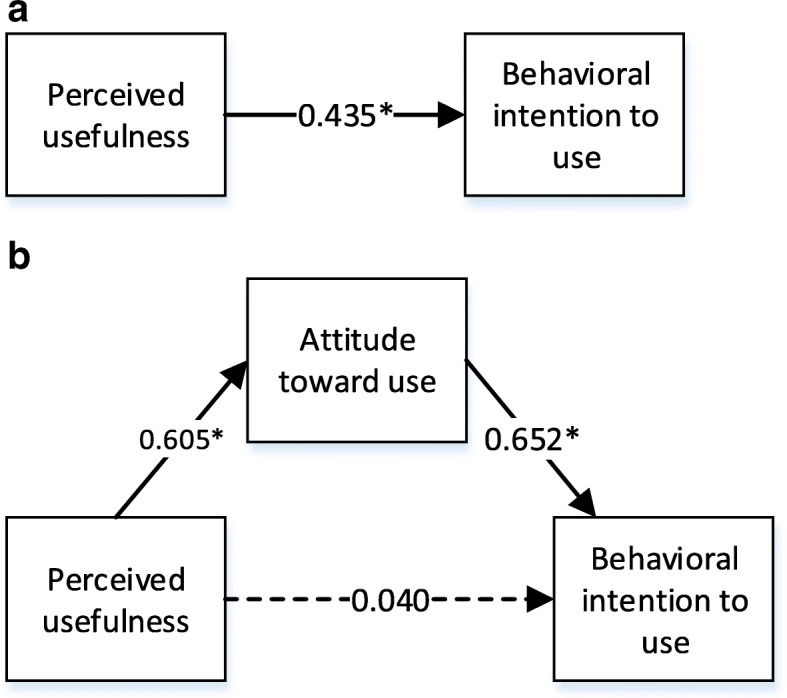
**a** Standardized parameters of direct effects. **b** Standardized parameters of indirect effects (mediation effect of attitude toward use)

Further comments for this research can be crucial to foster the whole concept. In this model, perceived usefulness and attitude toward use have intervening roles. In order to observe the relationships, new models are set up between perceived ease of use-attitude toward use and perceived usefulness-behavioral intention to use. So that, the role of perceived usefulness and attitude toward use will be seen in the implementation of extended technology acceptance model. According to the *p*-values provided in Figs. 3 and 4 there are significant relationships between perceived ease of use and attitude toward use, and perceived usefulness and behavioral intention to use, respectively.

Employment of PLS is to determine the effect of mediation, which is the strong relation between the predictor and criterion variable. In this paper, whether perceived usefulness and attitude toward use are the mediating variables or not are identified. As illustrated in Fig. 3, perceived ease of use has a significant direct effect on attitude toward use B-flow USG (β = 0.349, *p* < 0.05). When perceived ease of use and perceived usefulness are regressed on attitude toward use, perceived ease of use became insignificant (β = 0.016, *p* > 0.05), which is case in Fig. 3b. Hence, it can be concluded that perceived usefulness is a mediator.

Similarly, perceived usefulness has a significant direct effect on behavioral intention to use B-flow USG (β = 0.435, *p* < 0.05) (Fig. 4). When perceived usefulness and attitude toward use are regressed on behavioral intention to use B-flow USG, perceived usefulness became insignificant (β = 0.04, *p* > 0.05), which is case in Fig. 4b. Hence, it can be concluded that attitude toward use is a mediator.

## Discussion

The early period of a radiological assessment of intense abdominal torment in the pediatric age gather caused by the contorted pedicle in ovarian torsion stays testing [49]. In the mid-1990s, most specialists recommended that the visual evaluation of contorted adnexial structures and color change in the ovaries pictured by exploratory laparotomy or a laparoscopic strategy is the best quality level for the choice to continue with the medical procedure [4]. Be that as it may, it was proposed that visual finding amid laparoscopy could be misdirecting [1]. Today, it is concurred that discoveries of clinical assessment, ultrasonography and attractive reverberation imaging (X-ray) help choice to continue with the medical procedure. The patient history and the palpation of a mass in the lower abdominal segment may propose the likelihood of torsion, however ultrasonography is imperative for the differential determination from other clinical pathologies [1]. The assessment of the veins in the hilus locale is trying in the non-intrusive radiological examination.

The color Doppler is generally utilized for the ultrasonographic assessment of the ovarian tissue and the veins, and as of late, B-flow USG gave tasteful outcomes to the appraisal of blood flow rate and speed [49, 50]. The sonographic window and ancient rarities are constraints in the assessment of veins with color Doppler USG in the intense stage, however these impediments were overwhelmed by utilizing B-flow USG [7, 51, 52].

The proposed research model explains 45% of total variance of actual use of B-flow USG. A path analysis indicated that the majority results are consistent with the information system literature by showing that perceived usefulness, perceived ease of use, attitude toward use and behavioral intention to use are the significant determinants (direct or indirect) of actual use of B-flow USG. In addition to the TAM constructs, two external factors social influence and knowledge are also significant in predicting peoples’ actual use of B-flow USG.

This study contributes to recent research that found behavioral intention to use to be key determinant of actual use of B-flow USG. Next, it has been shown that social influence and attitude toward use are significant factors affecting the behavioral intention to use B-flow USG. Moreover, perceived usefulness has significant effect on attitude toward use B-flow USG. The results of the proposed model highlights knowledge and perceived ease of use as significant factors influencing perceived usefulness of B-flow USG.

The current research also shows that perceived usefulness is not affecting behavioral intention to use significantly; however, it affects behavioral intention to use indirectly attitude toward use. Similarly, perceived ease of use is not a significant determinant of attitude toward use B-flow USG; however, it affects attitude toward use indirectly through perceived usefulness. In addition, perceived usefulness mediates the relationship between perceived ease of use and attitude toward use, and attitude toward use mediates the relationship between perceived usefulness and behavioral intention to use B-flow USG.

### Implications

The findings of this research provide also useful inferences for stakeholders including B-flow USG users, hospital managers, investors, manufacturers and suppliers to recognize the perception toward the acceptance and adoption of B-flow USG. The decision makers need to recognize the constructs of the model that were tested in this paper.

The most noticeable aspect of this model is that social influence, knowledge, perceived ease of use, perceived usefulness, attitude towards use and behavioral intention to use are determinants of actual use B-flow USG. Among them, behavioral intention to use and social influence have the strongest impact on actual use of B-flow USG.

Social influence has been found to be a significant indirect determinant of actual use B-flow USG. The opinions of colleagues and peers may be very important among the users of B-flow USG. The physicians, hospital managements and medical communities can take an optimistic position toward system adoption. If the B-flow USG users are appreciated for their system use, the potential users might be motivated to use the it.

Another noticeable aspect of the results is that knowledge, perceived ease of use and perceived usefulness has indirect effects on intention to use B-flow USG, which indicates that users tend to rate B-flow USG useful if they have knowledge about and find easy to use it. In other words, physicians are likely to use B-flow USG more if they believe that using the system will increase their performance and productivity. Therefore, for B-flow USG to be successful, developers need to focus their attention on sufficient training programs, designing not only useful but also easy to use.

### Limitations and future research directions

Although the findings of the present study contribute to a better understanding of the factors that affect behavioral intention to use B-flow USG, there are several limitations to this study, which offers an opportunity for future research. Firstly, the current results cannot be generalized to the wider population due to the fact that the sample was obtained from physicians in Turkey. The findings might change if the model is retested in a different region. Any generalization of the results to other context should be made with caution although they are relatively reflecting the medical imaging technology. Secondly, demographic information that were collected through survey was not used as a factor in the proposed research model. As a future research, demographic characteristics including gender, age, education level or employment, can be incorporated in the model. Thirdly, even though some portion of the dependent variables are explained, 61% of behavioral intention to use is explained in the research model. Therefore, as a future research, further constructs may be taken into account that might be important in explaining behavioral intention to use B-flow USG. Lastly, this research can be integrated with qualitative study to understand and to explain the behavioral intention to use B-flow USG due to the fact that qualitative and quantitative aspects may complement each other to offer a better understanding.

## Conclusions

This paper aims to investigate the acceptance of a medical imaging technology, B-flow USG using the Technology Acceptance Model as the main theoretical framework, and integrate key determinants, including knowledge and social influence. A total of 528 surveys were collected from the medical doctors including radiologists, urologists, gynecologists, pediatric surgeons between June and October 2018, and 512 of the surveys were used in the analysis. So as to investigate the proposed research model, structural equation modeling using partial least squares methodology was conducted, which is widely used in several disciplines. SmartPLS software was utilized to analyze the collected data. This study enables both practical and theoretical implications for B-flow USG use. To the best of knowledge, this study is the first comprehensive study to integrate the constructs mentioned above to examine the antecedents of B-flow USG acceptance.

## Data Availability

The datasets used and analyzed during the current study are available from the corresponding author on reasonable request.

## Abbreviations

ATT: Attitude toward use
AU: Actual use
AVE: Average variance extracted
BIU: Behavioral intention to use
CFA: Confirmatory factor analysis
CR: Composite reliability
KNOW: Knowledge
PEU: Perceived ease of use
PLS-SEM: Partial least squares structural equation modeling
PU: Perceived usefulness
SEM: Structural equation modeling
SI: Social influence
TAM: Technology acceptance model
TRA: Theory of reasoned action
USG: Ultrasonography
